# Correction: Home bias and employee social responsibility: Identification vs. benefit exchange

**DOI:** 10.1371/journal.pone.0293220

**Published:** 2023-10-17

**Authors:** The PLOS ONE Staff

There are errors in the Funding section. The correct Funding statement is: This work was financially supported by the National Science Foundation for Young Scientists of China in the form of a grant (71902163) awarded to TZ. This work was also financially supported by the Humanities and Social Sciences Youth Foundation of Ministry of Education of China in the form of a grant (19YJC630218) awarded to TZ. This work was also financially supported by Guanghua Young Teachers’ Growth Plan of Southwestern University of Finance and Economics in the form of an award to TZ. This research was also financially supported by Fundamental Research Funds for the Central Universities in the form of a grant (JBK2202015) awarded to TZ. The funders had no role in study design, data collection and analysis, decision to publish, or preparation of the manuscript.

The publisher apologizes for the error.
