# Supplementary figures and images for: Home bias and employee social responsibility: Identification vs. benefit exchange
Source: PLoS One. 2022 Dec 8;17(12):e0278541. doi: 10.1371/journal.pone.0278541 (PMC9731460; doi:10.1371/journal.pone.0278541)

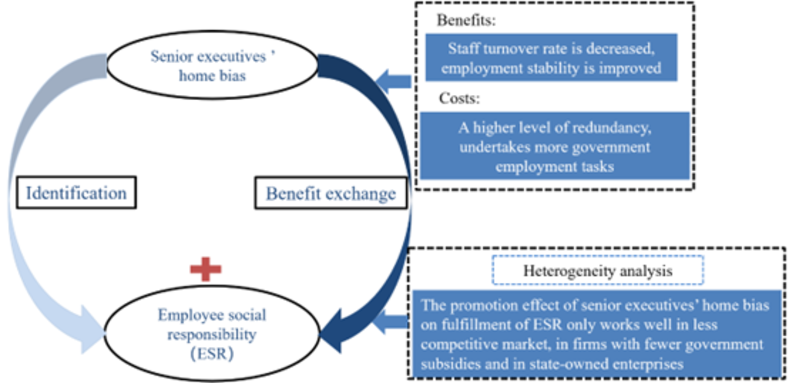

Supplement: S1 Graphical abstract — Both identification (left circle) and benefit exchange (right circle) are the motivation of this relationship, and benefit exchange is the more important motivation. The cost-benefit analysis and heterogeneity analysis further verify the benefit exchange motivation. (TIF) [file pone.0278541.s002.tif]
